# Genetic and epigenetic instability induced by betel quid associated chemicals

**DOI:** 10.1016/j.toxrep.2023.02.001

**Published:** 2023-02-04

**Authors:** Rajendra Bose Muthukumaran, Pritha Bhattacharjee, Priya Bhowmick, Lalrinawma Zote, Malsawmtluangi, Nachimuthu Senthil Kumar, Lalrintluanga Jahau, Marcus S. Cooke, Chiung-Wen Hu, Mu-Rong Chao

**Affiliations:** a Department of Chemistry, Mizoram University, Aizawl 796004, India; b Department of Environmental Science, University of Calcutta, Kolkata 700073, India; c Department of Biotechnology, Mizoram University, Aizawl 796004, India; d Centre for Rural Development Research and Trinity Diagnostic Centre, Aizawl 796004, India; e Oxidative Stress Group, Department of Cell Biology, Microbiology, and Molecular Biology, University of South Florida, Tampa, FL 33620, USA; f Department of Public Health, Chung Shan Medical University, Taichung 402, Taiwan; g Department of Occupational Safety and Health, Chung Shan Medical University, Taichung 402, Taiwan; h Department of Occupational Medicine, Chung Shan Medical University Hospital, Taichung 402, Taiwan

**Keywords:** Areca nut, Tobacco, Alkaloids, *N*-nitrosamines, DNA adducts, Epigenetic modifications

## Abstract

Over the years, betel quid chewing and tobacco use have attracted considerable interest as they are implicated as the most likely causative risk factors of oral and esophageal cancers. Although areca nut use and betel quid chewing may lead to apoptosis, chronic exposure to areca nut and slaked lime may promote pre-malignant and malignant transformation of oral cells. The putative mutagenic and carcinogenic mechanisms may involve endogenous nitrosation of areca and tobacco alkaloids as well as the presence of direct alkylating agents in betel quid and smokeless tobacco. Metabolic activation of carcinogenic *N*-nitrosamines by phase-I enzymes is required not only to elicit the genotoxicity *via* the reactive intermediates but also to potentiate the mutagenicity with the sporadic alkylations of nucleotide bases, resulting in the formation of diverse DNA adducts. Persistent DNA adducts provides the impetus for genetic and epigenetic lesions. The genetic and epigenetic factors cumulatively influence the development and progression of disorders such as cancer. Accumulation of numerous genetic and epigenetic aberrations due to long-term betel quid (with or without tobacco) chewing and tobacco use culminates into the development of head and neck cancers. We review recent evidence that supports putative mechanisms for mutagenicity and carcinogenicity of betel quid chewing along with tobacco (smoking and smokeless) use. The detailed molecular mechanisms of the extent of accumulation and patterns of genetic alterations, indicative of the prior exposure to carcinogens and alkylating agents because of BQ chewing and tobacco use, have not yet been elucidated.

## Highlights

• There are more than 600 million betel quid (BQ) chewers worldwide.
• BQ is often used with tobacco, either chewed or smoked.
• BQ use is one of the strongest risk factors for head and neck cancers.
• Potent mutagenic and carcinogenic substances in BQ are summarized.
• Both the genetic and epigenetic instability induced by BQ are firstly summarized.

## Graphical Abstract

**Figure:**
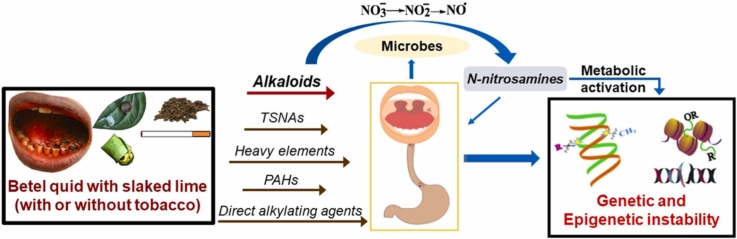

## 1 Introduction

As a result of rapid globalization, changing socio-economic status and attitude, lifestyle habits such as tobacco, areca nut or betel quid (BQ) use with or without tobacco has spread far and wide across the world among the adolescent and adult populations [1], [2], [3]. It is postulated that culturally accepted BQ use is the potential initiation stimuli for the tobacco use later [4]. Tobacco use and areca nut use confers higher risk for oral, esophageal, and stomach cancers [1], [3], [5]. The frequency and duration of BQ chewing, in a dose-dependent manner, is implicated for the development of oral leukoplakia, erythroplakia, and the concomitant oral cancer [6]. In fact, oral cancer incidence associated with BQ chewing and smoking was much higher in Taiwan than in South Asia by virtue of the number of BQs consumed per day as well as region-specific variations in BQ preparations [7]. In addition, alkaline BQ chewing without tobacco is implicated in the very high oral squamous cell carcinoma (OSCC) incidence in the New Guinean population which is also indicative of the carcinogenic potential of BQ and areca nut consumption [6], [8].

Alkaloids present in the lifestyle products such as areca nut, tobacco are susceptible to endogenous nitrosation in presence of other precursors under physiological conditions such as nitrite or nitric oxide which leads to the formation of nitrosamines [9]. The ensuing enzyme-induced metabolic activation of nitrosamines potentiates the carcinogenic and genotoxic attributes of nitrosamines. The metabolites of nitrosamines covalently bind with the DNA bases and form DNA adducts including methylated DNA bases [10], [11]. In addition, few DNA adducts are known to inhibit DNA repairing [9]. The accumulation of sporadic DNA adducts are responsible for aberrant mutations (G→A transitions and G→T transversions for nitrosamines of tobacco) which play an important role in the deregulation of cellular signalling pathways along with chromosomal instability and related carcinogenesis [9], [10]. The regulation of gene expression *via* epigenetic modifications, including DNA methylation and microRNAs, can control all pathways in the cellular network. Aberrant DNA methylations and dysfunctional histone modifications are the manifestations of exposure to various lifestyle risk factors and they can be considered as one of the important early biomarkers to predict the susceptibility of individuals to head and neck cancer [12], [13], [14]. We review recent genetic and epigenetic instabilities that strongly support putative mechanisms for the mutagenicity and carcinogenicity of BQ chewing along with tobacco (smoking and smokeless) use.

## 2 Areca nut and betel quid

**Fig. 1:**
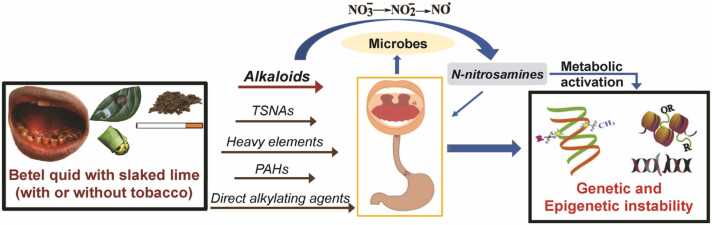
Overview of carcinogenic and genotoxic attributes of BQ chewing. Addictive lifestyle habits such as AN and BQ chewing (with or without tobacco) concurrently with smoking causes long-term oral exposure to alkaloids, nitrate, carcinogens (heavy elements, PAHs, *N*-nitrosamines) and alkylating agents. Oral microbial flora converts nitrate to nitrite. Nitrite in oral cavity is subsequently transformed to nitrosating species in mouth and stomach (stomach tissue in inflammation also releases inducible nitric oxide, a potential nitrosating species). The ensuing encounter between nitrosating species and alkaloids in oral cavity and stomach favorably enhances the endogenous formation of carcinogenic *N*-nitrosamines. In addition to the metabolic activation of *N*-nitrosamines, exposure to carcinogens and direct alkylating agents *in situ* induces sporadic genetic and epigenetic aberrations.

Areca nut (AN), the endosperm of the Areca palm (*Areca catechu)* fruit, is considered an important carcinogenic ingredient of different BQ preparations by virtue of its astringent polyphenols and potent alkaloids contents [15], [16], [17], [18]. Due to a multitude of ingredients employed for BQ preparations, and diverse combinations of BQ usage patterns render the assessment of relative risks of BQ chewing in different regions rather difficult [5], [7], [18], [19]. Although alkaloids constitute only as minor components in AN, they are important psychoactive and pro-carcinogenic chemical species. The major alkaloid arecoline can stimulate collagen synthesis with the activation of pro-collagen genes [20], [21]. BQ Chewing releases tannins and catechins into the oral cavity [15]. Tannins precipitate mucins in saliva that may lead to mucins depleted oral mucosa [16]. In addition, micro-nutrient elements including Cu^2+^ are released into the saliva during mastication and Cu^2+^ species can potentiate the cytotoxicity of AN extract by virtue of its’ synergetic interaction with organic components of areca nut [20]. The ensuing exposure to Cu^2+^ species and alkaloids in AN extract entails epithelial atrophy and chronic inflammation followed by progressive kertinization [22]. Concomitantly, chronic inflammation, a characteristic feature of OSF, activates the immune system to release cytokines. Many heavy elements are indeed co-carcinogens and they act synergistically in association with other carcinogenic species, thereby cause DNA damage (Fig. 1). Interestingly, under alkaline conditions (*vide infra*), Cu^2+^ species forms coordination complexes with the areca nut alkaloids and induce reactive oxygen species (ROS) generation [22]. Upregulation of transglutaminase 2, a Ca^2+^-dependent protein, catalyses protein-protein cross linkages leading to the BQ-chewing associated oral submucous fibrosis (OSF) by virtue of the intracellular ROS induced by arecoline [23]. Precancerous conditions such as OSF and oral lichen planus also causes hyposecretion of mucins. These conditions facilitate the penetration of small molecules into exposed oral mucosal cells and hence promote the development of oral cancer [16]. Oral mucosa cells, probably exposed in oral cavity of BQ-chewers, can metabolize the areca-specific alkaloids including arecoline, a possible carcinogen to humans [24], [25], [26]. Therefore, oral pre-malignant disorders such as leukoplakia, erythroplakia, OSF are the early manifestations of oral cancer [6].

Furthermore, addition of slaked lime as an alkaline agent to BQ serves two purposes: Firstly, it moderates the astringency arising from tannins of areca nut, and secondly, it facilitates the availability of ‘un-ionized’ alkaloids leading to the rapid absorption of arecoline in the oral mucosa [6], [25]. However, at high concentration levels, tannins and polyphenols also elicit pro-oxidant effect under alkaline conditions (pH ≥ 9.5) due to the addition of slaked lime and in presence of redox-active transition metals such as Fe^2+^, Cu^2+^ [27]. BQ containing AN and slaked lime (Ca(OH)_2_) with betel inflorescence generated significant levels of ROS than BQ containing AN and slaked lime with betel piper leaves. The generation of ROS is more likely due to the presence of polyphenols which elicit superoxide radicals by autoxidation in presence of Fe^2+^, and Cu^2+^ species [6]. When calf thymus DNA was incubated, under alkaline conditions, with an aqueous extract of AN *in vitro*, it also instigated the 8-oxo-7,8-dihydro-2′-deoxyguanosine (8-oxodGuo; so-called 8-OHdG) species formation. The BQ chewing induced oxidative stresses further corroborated by the observation of higher frequency of micronucleated oral epithelial cells of Indian BQ chewers with tobacco and slaked lime than the controls with no habits [6]. Under alkaline conditions (*vide supra*), the oral mucosal cells efficiently hydrolyse arecoline to yield arecaidine, while guvacoline is hydrolysed to yield guvacine. In addition, trace levels of N-methylnipecotic acid (NMPA), a nitrosylated metabolite of arecaidine is also detected in the BQ chewer’s saliva [25]. Arecoline is further metabolized to arecoline-N-oxide, and subsequently glutathionylated, as a part of detoxification pathway, to the corresponding mercapturic acid [28].

*In vitro* studies have shown that various AN products, *viz.*, unripe tender areca nut (used in Taiwan as a component of BQ), ripe and mature areca nut (consumed in Northeast Indian States of India), pan masala (a mixture of pre-packed finely chopped areca nut pieces, slaked lime, and condiments without tobacco), and gutkha (a mixture of pre-packed finely chopped areca nut, slaked lime, and condiments with tobacco flakes) [19] exhibit DNA alkylation potency in a dose-dependent manner. DNA alkylation potency of the aqueous extract of different areca nut products has shown the following order: gutkha > unripe tender areca nut > ripe and mature areca nut > pan masala [29]. This data commensurate with the arecoline contents in AN products, *viz.*, unripe areca nut>ripe mature areca nut>processed areca nut [4]. More importantly, highly polar chemical constituents of the aqueous extract of AN, which can be easily absorbed trans-orally, may be responsible for the DNA alkylation. As noted above, it is most likely that alkaloids and other polar compounds of tobacco and AN may augment the DNA alkylation potency of gutkha, in an additive manner rather than synergistic manner [28]. In addition, oral pre-malignant disorders (OPMDs), the manifestations of BQ as well as AN chewing, are also common in gutkha users [20].

Moreover, ROS inflicted DNA damage [30], probably, contributes to the development of carcinogenesis [31]. Importantly, with the downregulation of cell cycle inhibitors, *P21* and *P27 via* rapamycin complex-1 (mTOR) pathway and the upregulation of transcriptional repressor *Snail*, BQ-chewing induced increase in the intracellular ROS production results in the development of OSCC. Arecoline also plays an anti-apoptotic role with the inhibition of AMPK through ROS production [32]. The bio-activation of carcinogens by the enzyme-mediated phase-I metabolic processes play a major role in chemical carcinogenesis. Arecoline-N-oxide, an oxidative metabolite of arecoline exerted DNA damage and cytotoxicity, possibly, through the Cu^2+^ ion-mediated Fenton type reaction induced ROS production [27], [32]. Arecoline-N-oxide exposure also stimulates an elevated collagen expression and deposition *in vivo* [33]. It should be noted that significant levels of arecoline and arecoline-N-oxide (ARNO) were detected in OSCC tumor tissues [31]. ARNO elicits strong cytotoxicity effects, higher intracellular ROS levels, and depleted antioxidants and antioxidant enzyme levels, whereas a metabolite of arecoline and ARNO, *viz*., arecoline-N-oxide mercapturic acid ameliorates oral carcinogenesis [31].

## 3 Nitrate, nitrite, and endogenous nitrosation of AN alkaloids

After ingestion, nitrate is actively absorbed in the upper gastroinstestinal tract. In addition, modest amounts of nitrate can be endogenously formed as an oxidized end-product of the nitric oxide at the small intestine, by gut microflora [34]. Approximately 25 % of exogenous and endogenous nitrate reaches salivary glands through systemic circulation. Besides, lifestyle habits, *viz*., smokeless tobacco use, AN chewing, may supplement smaller amounts of nitrate in oral cavity [35]. At the oral cavity, oral microbiome reduces 10–90 % of nitrate to nitrite depending upon the oral hygiene (Fig. 1) [34]. The elevated endogenous nitrosation process in subjects with poor oral hygiene is more likely due to the bacterial enzyme-mediated reduction of nitrate to nitrite and the concomitant formation of *N*-nitrosamines in oral cavity [36]. At stomach, where mild alkaline saliva meets acidic gastric juice, nitrite is further reduced to nitric oxide (NO^•^) (Fig. 1) [34], [37]. In presence of nitrite-rich saliva, the acidic stomach also provides an ideal platform for the endogenous nitrosation process as it is an important site for S-nitrosation, N-nitrosation and O-nitrosation reactions [37]. Therefore, IARC designated that ingested nitrate or nitrite that result in endogenous nitrosation is *probably carcinogenic to humans* (Group 2 A) [38]. However, Nitrite reduction to nitric oxide in the stomach is strongly pH dependent and can be suppressed by high salt intake, proton pump inhibitor drugs as well as *Helicobacter pylori* infection that increase the gastric pH and also attenuates the ascorbic acid levels in gastric juice [39]. Moreover, consumption of fruits and vegetables enriched with antioxidants can inhibit the endogenous nitrosation, whereas ingestion of processed red meat rich in nitrite and high animal fat entails the elevation of nitrosation [38]. Tobacco products use, BQ use with or without tobacco, and high salt intake may act as the gastric mucosal irritants or damage the gastric mucosa and may also modulate the gastric acid secretion. It should be noted that high salt intake may accentuate carcinogenesis induced by other carcinogens [39], [40].

At neutral pH, entero-salivary nitrite is rather unreactive, albeit it becomes activated when pH < 4 [41]. Due to the presence of gastric juice refluxate at lower-esophageal segment (Barrett’s segment) during the acid reflux episode, ‘activated’ nitrite can be transformed into potent nitrosative species such as NO^**+**^, N_2_O_3_, as well as NOSCN (arising from salivary SCN^**−**^). In addition, with the ingestion of high-fat meal, lipid molecules can function as a “solvent” as they can dissolve and bring NO^•^ (generated from nitrite and inflammation induced iNOS) and O_2_ to close proximity, favourably enhance the formation of N_2_O_3_ [42]. These potent nitrosating species generate the endogenous *N*-nitrosamines when they encounter tertiary amine and secondary amine-containing alkaloids at Barrett’s segment in tobacco and BQ users (Fig. 1). Thus, gastric acid is an important etiological factor that causes the erosion and ulceration of squamous mucosa which results in reflux oesophagitis [34], [43]. With the erosion induced damage, due to gastric refluxate onslaught, luminal nitrosative chemistry occurs at Barrett’s esophageal segment, predisposing to adenocarcinoma [42], [43].

**Fig. 2:**
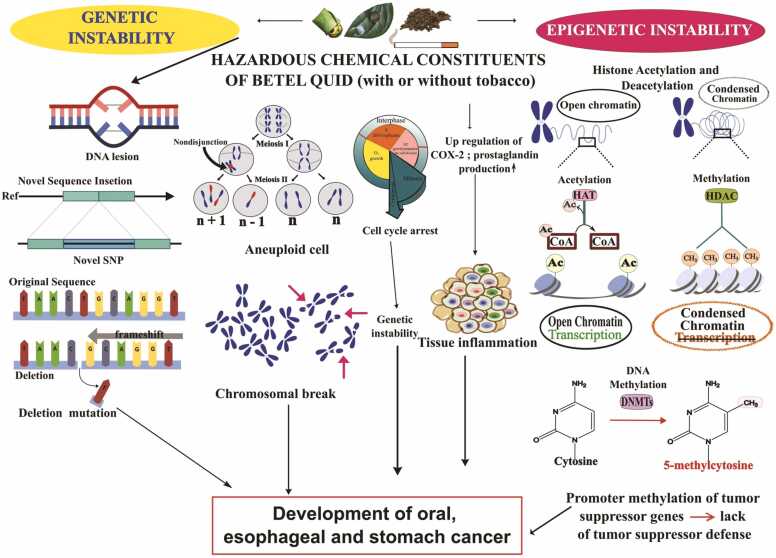
Induction of genetic and epigenetic instability due to betel quid chewing. Genetic instability includes formation of DNA adducts leading to deleterious DNA damage and promotion of chromosomal breakage. Besides, DNA mutations such as formation of novel SNP (Single Nucleotide Polymorphism), insertion-deletion mutation as well as the generation of aneuploid cells is also evident. Alterations in cell cycle pathways due to mutations and epigenetic modifications of certain cell cycle regulator genes can be observed. Epigenetic instability includes histone acetylation, methylation and promoter methylation of key regulatory genes involved in tumor suppressor defense, *i.e.*, normal cell cycle regulation. Promotor DNA hypermethylations of tumor suppressor genes leads to the upregulation of COX-2 *via* NFκβ pathway. Concomitant prostaglandins production reflects the inflammation and mucosal damage, which may eventually promote the development of oral, esophageal or gastric cancer.

More akin to nitrate, essentially derived from the diet, thiocynate (SCN^**−**^) is released in saliva, ranging from 0.1 to 2 mM, as a host defence against pathogenic microbes. However, in smoker’s saliva, the quantity of thiocyanate is significantly higher than a non-tobacco user by virtue of the exposure to hydrogen cyanide in tobacco smoke and concomitantly the depletion of glutathione is also observed [6]. The AN alkaloid-derived N-nitrosamines due to the exposure to AN alkaloids *via* BQ-chewing are 3-(*N*-nitrosomethylamino)-propionitrile (MNPN), 3-(*N-*nitrosomethylamino) propionaldehyde (MNPA), *N*-nitrosoguvacoline (NGCO) and *N*-nitrosoguvacine (NGCI) [6], [17], [44], [45]. Despite the knowledge on the formation of areca nut nitrosamines (ASNAs), a comprehensive understanding of the human phase-I enzymes that metabolize the ASNAs till remains to elucidated [31], [32], [45]. The current wisdom is that areca nut *N*-nitrosamines are metabolically activated *via* α-hydroxylation pathways mainly by the ubiquitous cyt-P_450_ systems [28], [45], [46]. Most *N*-nitrosamines in biological milieu are metabolically activated to exert their genotoxicity *via* the formation of reactive electrophilic intermediates. These transient electrophilic alkyl-azo-hydroxide species are potent alkylating agents that can induce G-C to A-T transitions (Fig. 2) [37]. Detoxification pathways involve phase-II enzyme(s)-mediated glucuronidation and flavin-containing monoxygenases catalysed N-oxidation of nitrosamines for TSNAs and ASAs [28], [46], [47].

## 4 Tobacco-specific nitrosamines

Nicotine, nornicotine, anatabine, anabasine are the major alkaloids of tobacco and precursors of potent carcinogenic tobacco-specific nitrosamines (TSNAs). Tobacco carcinogenicity is relatively well established and manifestations of tobacco carcinogenicity are also extensively studied [27], [47], [48], [49]. Overwhelmingly diverse types of smokeless tobacco products (STPs) with a wide range of carcinogenic properties are consumed worldwide. These STPs contain a wide range of compounds including alkaloids, nitrate, nitrite, TSNAs, and toxic contaminants such as pesticide residues, aflatoxins, and other mycotoxins [35], [50], [51]. Few of these chemical species can be classified by the IARC, based on their carcinogenic potential as Group 1 (aflatoxins, NNN, NNK, Cd^2+^), Group 2 A (nitrite, Pb^2+^), Group 2B (acetaldehyde, crotonaldehyde), and Group 3 (N′-nitrosoanatabine, N′-nitroso-anabasine) carcinogenic species as they are important determinants of oral, esophageal and stomach cancers [51].

TSNAs are biosynthesized during the curing and fermentation as a part of processing the tobacco matter. Microbial population at the surface of tobacco leaves effected the reduction of nitrate to nitrite. Concomitantly, TSNAs, *viz.*, N′-nitrosonornicotine [NNN], N′-nitrosoanatabine (NAT), N′-nitroso-anabasine (NAB), 4-(methylnitrosamino)− 1-(3-pyridyl)− 1-butanone (NNK), 4-(methylnitrosamino)− 4-(3-pyridyl)-butanal (NNAL), 4-(methylnitrosamino)− 4-(3-pyridyl)− 1-butanol (iso-NNAL), and 4-(methylnitrosamino)− 4-(3-pyridyl)butyric acid (iso-NNAC) are formed during the curing, aging and the processing of tobacco leaves [52]. Interestingly, nitrosamines are also formed due to the interaction of transition metal ion, Cu^2+^ with alkaloids in tobacco [53]. Importantly, TSNAs are volatilized and also formed *de novo* (in moderate levels) during the combustion of tobacco matter in smoking tobacco products [54]. Metabolism of some TSNAs potentiates the carcinogenicity of TSNAs. Among the TSNAs, NNN, NNK, NNAL are strong carcinogenic species (IARC group 1 carcinogen), while NAT and NAB are weakly carcinogenic, whereas iso-NNAL, iso-NNAC are negatively associated with carcinogenicity [49]. In animal model studies, it is established that NNN as an oral and esophageal carcinogen, and NNK as a lung carcinogen. NNAL is known to elicit lung, liver and pancreatic tumors [49]. Importantly, NNN is the preponderant *N*-nitrosamine species along with nitrate ion in STPs available in South Asia [35], [48], [55] albeit NNN is most likely an oral and esophageal carcinogen in humans [10]. NNN absorption due to STPs use may be correlated with high esophageal cancer incidence in India (malignancy location is preponderantly at the mid and distal esophagus, and 79 % dominated by squamous cell carcinoma) with the male to female ratio of 1.67 [56], [57].

Carcinogens can be metabolically transformed, in a diverse manner, either as metabolically activated metabolites or detoxified and eliminated [10], [52]. Metabolically activated products can exert carcinogenesis as they act, in multiple ways, through the formation of DNA and protein adducts or through the generation of ROS [32], [52]. Secondary amine or tertiary amine containing alkaloids undergo nitrosation reactions and the N-atoms of these alkaloids are attacked by the electrophilic nitroso compounds and the formation of N-N bonds on alkaloids gives rise to *N*-nitrosamines [58]. Chronic use of alkaloids rich lifestyle products such as tobacco or AN provides a facile environment for the endogenous formation of *N*-Nitrosamines in the metabolic redox milieu (Fig. 1) as well as exposure to exogenously formed *N*-nitrosamines [54]. Concomitantly, the metabolic activation of N-nitrosamines potentiates their respective carcinogenic activities [49]. Metabolic activation of *N*-nitrosamines generates electrophiles which can effectively interact with DNA and proteins of important cellular pathways to form DNA and proteins alkylation adducts to initiate the carcinogenesis process [11].

Metabolic activations of tobacco-specific *N*-nitrosamines such as NNN and NNK lead to the formation of unstable electrophilic diazohydroxide intermediates, concomitantly transformed to short-lived alkyl-diazonium ion intermediate species [47]. These unstable electrophilic species attack nucleophilic sites of DNA, RNA, and protein systems to form the corresponding adducts (Fig. 2). The activated form of *N*-nitrosamines, by S_N_1-nucleophilic substitution, induce various DNA adducts [59], and DNA-RNA crosslinks [60]. The formation of DNA adducts provides the impetus for somatic mutations [47], [49]. The accumulation of genetic and epigenetic abnormalities constitutes carcinogenesis.

An increase in the frequency of adenocarcinoma of distal esophagus and gastric cardia is generally observed by virtue of high dietary intake of nitrate along with processed meat products, whereas squamous cell cancer is prevalent in population with nutritional deficiencies including reduced water soluble vitamin intake [34], [38]. Chronic *H. pylori* colonization and the concomitant exposure to *H. pylori* toxins lead to atropic gastritis [39]. Moreover, chronic *H. pylori* infections could contribute to the development of distal gastric cancers due to attenuated gastric acid and ascorbic acid secretions. Furthermore, *H. pylori* infections also modulate the promotor methylation [39]. In fact, the exposure to *H. pylori* toxins is characterized by gastric oxidative stress, reactive aldehyde formation, cellular RNA and DNA damage, DNA promoter genes hypermethylation, persistent mucosal inflammation, and achlorhydria [40]. *H. pylori* infection is the one of the important causal factors of distal gastric adenocarcinoma [61]. In Northeast India (NEI), esophageal and stomach cancers depicts more pronounced male preponderance [61], [62]. Moreover, induction of neutrophil myeloperoxidase and macrophage Ca^2+^/calmodulin dependent nitric oxide synthase (NOS) enzymes and the associated ROS pathways involving hydrogen peroxide is also observed. Interestingly, carcinogenic nitrosamines are formed in gastric mucosa when inducible nitric oxide synthase activity is increased along with attenuated gastric mucosal ascorbic acid levels. This synergistic epithelial oxidative stress, deregulated promotor methylations and the attenuated antioxidative capacity portend the promotion of carcinogenesis [39], [40].

## 5 Betel quid and its associated genetic instability

Areca nut (AN) is an essential constituent of BQ and is declared as a group I carcinogen by the International Agency for Research on Cancer (IARC) [18], [48]. However, the molecular and cellular mechanisms regarding the carcinogenicity of AN are not fully understood. Genomic instability is one of the hallmarks of cancer that is associated with disease progression [63]. Arecoline is not genotoxic albeit cytotoxic *in vitro* and tumorigenic *in vivo* [64]. However, AN alkaloids may be important environmental risk factors for OSCC [65]. Abnormalities in the genetic and epigenetic factors induced by long-term BQ chewing cumulatively influence the development of head and neck cancers [44], [66]. In addition, due to the presence of hazardous substances (arecoline, aflatoxin B1) in fermented AN, changes in numerous biological pathways, including aberrant gene expressions associated with xenobiotic metabolism, chromosomal structural integrity, and DNA repair mechanisms, have been reported. Toxicity of arecoline was linked to novel point mutations of *AR*, *BRCA1, IL8*, and *TP53* genes. Aflatoxin B1 has been linked to the *BARD1, BRCA2, CCND2, IGF1R, MSH6,* and *RASSF1* genes with novel deletion as well as the *APC, BRMS1, CDK2AP1, CDKN2B, GAS1, IGF1R*, and *RB1* genes with novel insertion [67].

In OSCC patients, BQ-derived carcinogens may encourage DNA damage that facilitates genomic instability. BQ-related OSCC is linked to somatic aberrations such INDELs (insertion/deletion mutations), SV-related breakpoints, and certain mutational signatures (signatures 1, 7, and 13). In cases of oral mucosal epithelial malignancies, BQ users have a higher number of INDELs than non-BQ users. Besides, compared to non-users, in BQ-users a greater number of chromosomal aberrations are also observed. The greater prevalence of C>T substitutions in tongue carcinomas is linked to habitual BQ chewing [68]. Aqueous extract of raw mature areca nut (RAN) stimulates stomach cancer [69]. By virtue of persistent RAN consumption with slaked lime, larger amounts of precocious anaphase and aneuploidy cells (Fig. 2) were detected in the bone marrow cells. Overexpression of p53, Bax, Securin, and p65 are observed in the stomach and esophageal cells (Table 1) [69].

**Table 1:** BQ and its associated genetic instability.

Model	Dose/Exposure	Biological Sample	Method	Observation	Ref
N = 25 (patients of oral cancer)	Habitual chewers	Tissue samples collected from endoscopic biopsy	1. Next Generation sequencing 2. Comparative Toxogenomic database tool (for the analysis of association between toxic compounds present in BQ and oral cancer)	▪ Alteration of various biological pathways such as alteration of genes involved in xenobiotic metabolism, chromosome structural stability, DNA repair mechanism observed due to the presence of toxic compounds in BQ. ▪ Novel SNP of *AR, BRCA1, IL8,* and *TP53* were found to be associated with arecoline. ▪ Novel SNP of *ADH6, APC, AR,* ▪ *BARD1, BRMS1, CDKN1A, E2F1, FGFR4, FLNC, HRAS, IGF1R, IL12B, IL8, NBL1, STAT5B*, and *TP53* were found to be associated with aflatoxin B1. ▪ Genes *BARD1, BRCA2, CCND2, IGF1R, MSH6,* and *RASSF1* with novel deletion and genes *APC, BRMS1, CDK2AP1, CDKN2B, GAS1, IGF1R,* and *RB1* with novel insertion were found to be associated with aflatoxin B1.	[67]
N = 196 male patients with OSCC (N = 95 habitual BQ chewers, N = 101 non-BQ users)	chewing one or more BQ-related products daily for at least 1 year	Fresh-frozen tumors and matched whole blood	1. Exome sequencing 2. Detection of Single Nucleotide Variation and Insertion/ Deletion 3. Analysis of mutational signatures	▪ BQ-derived carcinogens may promote DNA lesion facilitating genomic instability in OSCC patients. ▪ Somatic abberations such as INDELs, SV-related break-point, specific mutational signatures (signatures 1,7 and 13) are associated with BQ-related OSCC. ▪ Higher number of INDELs are observed in BQ users compared to non-BQ users in case of cancers of the buccal mucosa. ▪ Higher number of chromosomal breaks are detected in case of BQ-users. ▪ Signature 1 revealed higher frequency of C>T substitutions in BQ-related tongue carcinoma. ▪ An additional signature 5 has been detected in BQ-related tumors.	[68]
3 groups of (Swiss albino mice) mice (N = 25 in each) and another 3 groups of mice (N = 15 in each) for tumor induction studies	RAN-extract *ad libitum* in drinking water with or without lime (1 mg/day for first 60 days, subsequently2 mg/day till 120 days)	Bone marrow cells, esophageal cells and stomach cells	1. Real-time RT-PCR 2. Immunoblotting	▪ RAN induces stomach cancer. ▪ **↑** of precocious anaphase and aneuploidy cells was observed in the bone marrow cells with a greater intensity in RAN+ lime intake. ▪ **↑** levels of *p53*, *Bax*, *Securin* and *p65* in esophageal and stomach cells ▪ **↓** of Aurora kinases, *Mad2*, *Bub1* in bone marrow, esophageous and stomach cells of the mouse. ▪ Apoptotic cell death was observed in non-cancerous stomach cells but not in tumpor cells.	[69]
N = 10,657 participants (all male patients age ≥ 18 years) [ranged from 18 to 96 years with an average age of 55.2 years (*±*18.6 years)]	Habitual chewers	Oral inspection of participants	1. Real-time RT-PCR 2. Bivariate analysis 3. Logistic Regression model	▪ 514 (4.8 %) participants were recorded to have positive lesions and among them 344 (66.9 %) participants underwent oral cavity biopsy. Among those 230 (66.9 %) participants were proven to have oral cancer. ▪ Those who have 3 habits (smoking, alcohol consumption, BQ chewing) were most likely to develop cancer. (odds ratio: 46.87, 95 % confidence interval: 31.84–69.00)	[70]
N = 100 BQ-chewers N = 100 non-BQ chewers N = 100 oral cancer patients	Habitual chewers	1. Buccal cells 2. Peripheral blood samples	1. Real-time RT-PCR 2. Data analysis of STR markers	▪ In case of oral cancer patient 2 types of DNA instability were observed (allelic alterations including the expansion and contraction). ▪ 2 loci with major allelic imbalance were detected in 100 healthy BQ chewers.	[71]
Cell line - Gingival Keratinocyte (Human GK)	Areca Nut Extract (200–800 µg/ml) (24 h exposure)	Human GK (Gingival Keratinocyte)	1. Real-time RT-PCR 2. Immunoblotting	▪ **↑** of prostaglandin production by 1.4–3.4 fold following exposure to 200–800 µg/ml of AN extract for 24 h. ▪ Cell retraction and intracellular vacuole production are observed. ▪ At **↑** concentrations, AN extract induced cell death. ▪ AN extract stimulates oral submucous fibrosis and oral cancer *via* stimulatory effect on PGs, COX-2 production and associated tissue inflammation.	[73]
Cell line-Normal Oral Human Keratinocyte (NHOK)	Ripe Areca Nut Extract (ANE)	Normal Oral Human Keratinocyte (NHOK)	1 Real-time RT-PCR 2. EMSA 3. Western blot analysis 4. 5-bromo-20-deoxyuridine (BrdU) incorporation assay 6. Senescence-associated b-galactosidase (SA-b-Gal) assays 7. Combined SA-b-Gal and BrdUlabeling assay 8. Flow Cytometry	▪ ANE **↓** cell viability and proliferation ▪ ANE **↑** G1 arrest and apoptosis ▪ **↑** of p^16^ and p^21^ ▪ **↑** senescence-associated β-galactosidase ▪ Genomic instability is observed during long-term ANE treatment ▪ **↑** level of nuclear NF-kB ▪ upregulated IL-6 and cyclooxygenase-2 ▪ (COX-2) mRNA expressions in late-passaged NHOK are also evident.	[74]
N = 35 Swiss albino mice N = 32 humans	RAN extract *ad libitum* in the drinking water with lime (pH 9.8) Initially 1 mg extract/day for 60 days then the dose was increased 1–2 mg/day till 120 days	Mouse bone marrow cells and peripheral blood from human donors	1. Immunobotting 2. Histopathological evaluation 3. Western blotting	▪ **↑**of precocious anaphase and aneuploidy cells was observed in both RAN+lime treated mouse BMC and human PBL of heavy RAN consumers. ▪ Stomach tissue of mice developed RAN+lime induced stomach cancer. ▪ **↑**levels of p^53^ and securin are observed which is related to chromosome instability and associated carcinogenesis.	[75]
Cell line (HEp-2 and KB)	Arecoline, a major alkaloid of areca nut (0.3 mM arecoline)	HEp-2 (Human larynx epithelioma cancer) and KB (Human epithelial carcinoma) cell line	1. Real-time RT-PCR 2. Western blotting 3. Flow cytometry 4. In vitro microtubule assembly assay	▪ Arecoline stabilizes mitotic spindle assembly leading to distorted organization of mitotic spindle. ▪ Arecoline also induces misalignment of chromosomes and ↑ of spindle assembly checkpoint genes (SAC) leading to prometaphase arrest. ▪ Arecoline causes group of cells containing various chromosomal abberations and exhibited genomic instability.	[76]
Young male and female Swiss albino mice	aqueous extract of AN(AEBN) in drinking water (2 mg.ml−1) for up to 24weeks for chronic exposure and	Whole homogenates of liver, spleen cells, enlarged lymph nodes, pus-filled sacs and solid tumors	1. Slot blot and Western immunoprobing 2. Imaging and densitometric analysis 3. Molecular modeling of predicted protein sequences 4. DNA sequencing	▪ **↓**in levels of *Brca1*, *Brca2, p*^*53*^ indicating a loss of tumor suppressor protection. ▪ Mutation in exon 11 of *Brca1* gene increased the risk of cancer.	[77]
N = 116 HNSCC	Habitual chewers	Squamous cell carcinoma and peripheral blood	1. Multiplex PCR 2. Capillary array electrophoresis	▪ More than half of the specimens (n = 68, 58.6 %) had loss of heterozygosity (LOH) in at least one marker, while 44 specimens (37.9 %) had at least one marker with microsatellite instability (MSI).	[78]

Individuals who have the habits of smoking, alcohol consumption and BQ chewing were at higher risk of developing oral cancer (odds ratio: 46.87, 95 % confidence interval: 31.84–69.00) [70]. In oral cancer patients, two forms of DNA instability (allelic alterations including the expansion and contraction) are also observed (Table 1) [71]. Arecoline potentiates collagen synthesis with the activation of TGF-β pathway that causes OPMDs [20]. Under oxygen depleted environments *in vitro*, HIF‐1α enhances epithelial–mesenchymal transition (EMT) and promotes fibrogenesis with the overexpression of extracellular matrix‐modifying factors and lysyl oxidase genes leading to the formation of OSF that may be attributed to BQ or areca nut chewing [72]. In addition, through its stimulatory action on prostaglandins, COX-2 production, and related tissue inflammation, ripe areca nut extract (ANE) promotes OSF and oral cancer in Human GK (Gingival Keratinocyte) (Fig. 2) [73]. ANE induces G1 cell arrest by upregulation of *p16* and *p21* expression in NHOK (Normal Oral Human Keratinocyte). It is also clear that late-passaged NHOK exhibits elevated *IL-6* and *COX-2* mRNA expressions [74].

When treated with RAN and slaked lime, mouse bone marrow cells (BMC) as well as human PBL of heavy RAN users exhibited high levels of premature anaphase and aneuploidy cells. Securin and p53 proteins were found in high concentrations, indicative of chromosome instability and related carcinogenesis. Besides, cancerous tumor also appeared in mouse stomach tissue by the stimulatory action of RAN and slaked lime [75]. Arecoline stabilizes the mitotic spindle assembly, causing erratic organization of the mitotic spindle. Additionally, it causes chromosome misalignment and the deregulation of spindle assembly checkpoint genes (SAC), which induces prometaphase arrest in HEp-2 cell line [76]. Choudhury et al., observed the repression of *BRCA1, BRCA2*, and *p53* that signify the lack of tumour suppressor defence. Consequently, the likelihood of developing cancer was significantly increased by the presence of mutation in exon 11 of *BRCA1* in Swiss albino mice [77]. Microsatellite changes were evident in head and neck squamous cell carcinoma (HNSCC) patients with BQ chewing habit (Table 1) [78].

## 6 Genetic instability due to smokeless tobacco products use

By virtue of the hydrophilic and direct acting DNA alkylating agents present in tobacco matter, long-term STPs use portends adverse oral health effects [29], [79]. In India, the world’s second-largest tobacco consumer, where predominantly STPs are used extensively by > 200 million people and STPs act as a putative causative factor of various cancers [80], [81]. Some states of NEI have the highest prevalence of STPs use such as Mizoram has the highest prevalence of STPs use (47.8 ± 1.2) followed by Manipur among females (46.1 ± 0.7) (by National Family Health Survey-4, NFHS-4) [82]. Most popular STPs include khaini, gutkha, zarda, paan or BQ with tobacco, loose tobacco leaf or sadapata, tuibur or hidakphu [81]. STPs can exasperate the risk of various cancers, *viz*., oral cancers, pancreatic cancer, oesophageal cancer, gastric cancer [83]. STPs contain diverse carcinogens, including alkaloids (nicotine, nornicotine, anabasine *etc.*), carbonyl compounds (acrolein, crotonaldehyde), nitrosamines (NNN, NNK, NAB, NAT), polycyclic aromatic hydrocarbons (PAHs) and heavy elements (As, Pb, Cd, Cu, Hg, U, Po *etc.*). Chronic STPs use is also an important etiological factor for the development of many tobacco related disorders including cancer [81], [84], [85], [86].

Nicotine acts through Cyclooxygenase 2 dependent pathway [87]. It binds β adrenergic receptors and promotes cell survival and cell growth as well [88]. Nicotine undergoes peroxidation and forms myosmine that releases activated methyl-species and could methylate DNA and protein [89]. Nornicotine binds to nicotine acetylcholine receptor (AchR) activates signaling pathways that result in increased cell proliferation and cell survival. Rapid Akt activation and suppression of apoptosis is also reported. Nicotine and nornicotine act through NF-κβ and MAPK signalling pathways in case of gastrointestinal carcinogenesis. Arecoline and guvacoline also stimulate prostaglandin production and COX2 expression.

Metabolic activation of NNK and NNN by α-hydroxylation generates DNA methylating and pyridyl-oxobutylating intermediates that can cause deleterious mutations by forming DNA adducts in oncogene and tumour suppressor genes [11]. Novel SNPs in *ATM*, *BRCA1*, *CDKN1A*, *EGFR*, *IL8*, and *TP53* genes of NEI population is linked to the TSNAs toxicity [67]. In oral and esophageal carcinoma, FAK/Src complex phosphorylation promotes MEK1/2 and ERK1/2 (MAP kinase family) phosphorylation [90]. This signal transduction cascade leads to enhanced MMP-2/MMP-9 protease secretion and the downregulation of *TIMP* which degrade extracellular matrix and cause cellular membrane disruption. Thus, overexpression of MMPs is directly correlated with cancer cell invasion and metastasis [44], [90], [91].

Nicotine also diminishes epithelial β-catenin and E-cadherin levels and concomitantly increases the mesenchymal proteins fibronectin and vimentin levels through the activation of PI3/AkT pathway. Thus, nicotine elicits EMT leading to the disruption of cell–cell adhesion. This modulation of different cellular cascades by nicotine more akin to arecoline renders the cell to acquire migratory and motility properties which may promote metastasis [72], [89], [92]. With the upregulation of MMPs, COX-2, VGFR, urokinase-type plasmogen activator (uPA), nicotine also promotes pro-angiogenic activity. Besides, a mimick of nicotine, nornicotine can be nitrosylated to form NNN, a human carcinogen, in saliva [89]. As a surrogate of nicotine, NNK binds to α_7_-nicotinic acetylcholine receptor and elicits the influx of Ca^2+^ as well as the release of serotonin which leads to the activation of protein kinase cascade (PKC/Raf-1/MEK/ERK1/2). As noted above, NNK stimulates the simultaneous phosphorylation of major oncoproteins, Bcl-2 and c-myc. Consequently, the direct interaction between Bcl-2 and c-myc deregulates multiple functions of c-myc protein such as the inhibition of apoptosis while promoting the development and proliferation of neoplasms [93]. Mutant copies of onco-proteins EGFR, K-ras, c-myc, and cyclin D1, have been implicated in the development of OSCC [94]. Miscoding of DNA due to DNA adduct formation in *p53* leading to GC→AT transition mutations are also observed. Overexpression of aberrant p53 mutant proteins arising from these mutations are often detected in tumor tissues [95]. Specific *p53* codon polymorphisms were implicated for the susceptibility to OSCC due to tobacco, BQ, alcohol use and HPV infection [96]. In fact, chronic HPV infection is causally linked to the development and progression of oro-laryngeal malignancy [44], [94]. Besides, these abnormal p53 proteins may promote cell migration, invasion and the concomitant metastasis with the induction of oncogenes and repression of tumor suppressor genes [44], [94], [95], [97].

## 7 Betel quid and its associated epigenetic instability

In South and Southeast Asia, areca nut use is a significant etiological factor for oral cancer, which is also the fourth most prevalent malignancy in men in Taiwan [98]. BQ chewers who also smoke, and consume alcohol, have a 123-times more oral cancer risk than non-users [99], [100]. The gene inactivation of methyl transferase, MGMT, by promotor hypermethylation, possibly plays a key role in the tumorigenesis, especially when exposed to direct DNA alkylating agents through chronic lifestyle habits, causing chromosomal aberrations (Fig. 1) [52], [101]. Penetration of small molecules into exposed oral mucosal cells due to AN chewing alters the epigenome during the development of cancer [44], [66]. DNA methylation prevents transcription factors from binding to DNA either directly by "masking the DNA" or inadvertently by attracting methyl-CpG binding proteins (MBPs), which have repressive chromatin-remodeling properties [66]. The majority of histone modifications, including acetylation, methylation, phosphorylation, ubiquitination, and SUMOylation, takes place in their unstructured, alkaline N-terminal tails. The regulation of chromatin structure by these post-transcriptional modifications has an impact on biological processes like gene expression, DNA repair, and chromosomal condensation (Fig. 2) [66]. It has been demonstrated that heavy metal ion cadmium, present at trace levels in lifestyle products (*vide supra*) [102], [103], can induce global DNA hypermethylations with the upregulation of DNMT genes (Fig. 1) [12], [79]. The development of OSCC is most likely the manifestation of numerous accumulated genetic and epigenetic alterations.

Carcinogens induce epigenetic alterations in oral mucosal cells and the long-term stimulation of those cells due to lifestyle habits such as AN or BQ chewing and tobacco use can cause oncogenes and tumour suppressors including *p53*, *BRCA2*, and *XRCC4* to express abnormally [104], [105], [106], [107], [108]. Oral cancer and esophageal cancer are the two cancers that are most frequently linked to consuming areca nut. The comparison of aberrant mutations (C→A) as well as the methylation changes of *TFAP2E* in esophageal squamous cell carcinoma (ESCC) and the nearby healthy esophageal mucosal cells of Taiwanese ESCC patients may possibly be indicative of esophageal cancer risks [109]. Animal studies have shown that 3-(methylnitrosamino)-propionitrile (MNPN), a carcinogen detected in areca nut chewers' saliva, induces DNA methylation in the liver, oesophagus, and nasal mucosa [110]. Since *H3K9* methylation is essential for preserving the stability of heterochromatin structures and suppressing euchromatic gene expression, a reduced amount of *H3K9* methylation after arecoline induction impairs chromosome stability in leukemia K-562 cells (Table 2) [111].

**Table 2:** BQ and its associated epigenetic instability.

Model	Dose/Exposure	Biological Sample	Method	Observation	Ref
Cell line (Human K-562 cells)	Cells were incubated with different concentrations of ARC (Arecoline) (0, 200, 400, 800, or 1600 μg*/*ml) for 24 h.	Human leukemia K-562 cells	1.Cytotoxicity Assay 2.Real-time RT-PCR 3.Western blotting	• **↓**of cell viability in different concentrations of ARC. • Involved in oral cancer as well as hepatocellular carcinoma. • ARC suppressed *Suv39h2* expression which is associated with genomic instability and increased risk of B cell lymphoma. • **↓** of *H3K9* methylation following ARC induction disturbs chromosome stability since is involved in maintaining the stability of heterochromatin structures and inactivating euchromatic gene expressions**.**	[111]
**N = 74** patients (39 males, 35 females) N = 24 BQ chewers with OSCC, N = 25 BQ non-chewers with OSCC, N = 25 nonchewing healthy control subjects.	Habitual chewers	OSCC and normal oral mucosa tissue samples	1. Quantitative methylation-specific PCR 2. Real-time quantitative reverse-transcription PCR 3.Western blotting	• DNA hypermethylation of tumor suppressor genes is observed in precancerous lesions and oral cancer of individuals. • **↑** levels of methylation of *SIRT1* was significantly ↑ in OSCC of patients with BQ chewing habits than in BQ non chewers (p < 0.05) leading to oral carcinogenesis.	[14]
N = 48 resected primary oral cancers and nearby non- cancer tissue samples	Habitual chewers	OSCC	1. Methylation specific PCR 2. Sequencing analysis	• Epigenetic alteration of tumor suppressor genes *p*^*15*^*, p*^*16*^*,p*^*53*^*,VHL*isobsereved. • The frequencies of aberrant methylation on the promoter of the *PI5, P16,P53* and the *VHL* genes were 0.27 (13/48), 0.42 (20/48), 0.04 (2/48) and none respectively. • The inactivation of these genes isa prerequisite for carcinogenesis.	[112]
N = 93 patients ESCC	Raw AN (RAN) chewing (N = 34 are only RBN-chewing habit)	ESCC	1. PCR 2. Methylation specific-PCR	• In 40% of ESCC samples with RBN-chewing alone, loss of the microsatellite markers D9S1748 and D9S1749, which are adjacent to exon 1 of the *CDKN2A/ARF* gene at 9p21, was observed. • Samples with RBN alone chewinghad significantly higher promoter hypermethylation of the *CDKN2A* gene (p = 0.01).	[114]
N = 64 patients (oral pre-cancerous)	Habitual BQ chewers	Tissue samples	1.Methylation specific-PCR 2.Immunohisto-chemical staining	• Although there was no hypermethylation observed in normal epithelium, pre-cancerous lesions had a significant incidence of *p14*, *p15*, and *p16* hypermethylations. • *p14, p15*, and *p16* hypermethylation occur whether or not the lesions have *p53* mutations because the hypermethylation was highly detectable even in *p53*-negative lesions.	[115]
N = 6 groups of mice (Swiss albino mice)	RAN (Raw Areca Nut) For 60 days, drinking water was treated with 1 mg of RAN extract with lime daily, *ad libitum*, and then the dosage was raised by 1 mg every 60 days following that.	Stomach tissue	1. Histopathological evaluation 2. Immunoblotting 3.Immunohisto-chemistry 4. ChIP-qPCR assays	• After 300 days of RAN feeding, *securin* overexpression manifested in all mice as stomach cancer. • In the RAN-treated samples, immunohistochemistry results showed that Rb was hyperphosphorylated and that *E2F1* was upregulated. • Following RAN treatment, higher amounts of lysine-N-methyltransferase 2 A, lysine-acetyltransferase, EP-300, and PCAF were found, which led to an increase in the trimethylation of H3 lysine 4 and acetylation of H3 lysine 9 and 18 both globally and in the promoter region of the securin gene.	[116]

Previous studies have shown that BQ chewing alters the expression of various genes involved in histone methylation (*MII, Setdb1*, and *Suv39h2*), acetylation (*Atf2*), and demethylation (*JMJD6*) [110], [111]. *Retinoic acid receptor β* (*RARB*) gene promotor hypermethylation and the concomitant repression of *BARB* plays an important role in the BQ chewing related OSCC progression [100]. BQ chewing also results in DNA hypermethylation of sirtuin 1 (*SIRT1*), which may be a putative biomarker for malignant transformation. Individuals with oral cancer and precancerous lesions have DNA hypermethylation of tumour suppressor genes, and higher levels of *SIRT1* methylation were found in OSCC patients who chewed BQ more frequently than those that did not (p 0.05), which contributed to oral carcinogenesis [14]. Aberrant methylation on the promoter of the *P15, P16, P53* and the *VHL* genes are observed in habitual chewers [112]. Importantly, epigenetic changes including differential alternative splicing of mRNA precursors and insertion/deletion polymorphisms are the major aberrant mechanisms of *P53*, possibly due to BQ chewing [95], [113].

In previous studies, *CDKN2A* had considerably higher promoter hypermethylation in the patients with ESCC who have chewed RAN (raw areca nut) only (p = 0.01) [114]. Normal epithelium did not have any hypermethylation, whereas pre-cancerous lesions exhibited *p14*, *p15*, and *p16* hypermethylation. Since, the hypermethylation was clearly visible even in *p53*-negative lesions, it is clear that *p14, p15*, and *p16* hypermethylation takes place whether or not the lesions carry *p53* mutations [115]. Overexpression of lysine-N-methyltransferase 2A, lysine-acetyltransferase, EP-300, and PCAF enzymes were observed after RAN treatment, which resulted in an increase in the trimethylation of H3 lysine 4 and the acetylation of H3 lysine 9 and 18 both globally and in the promoter region of the *securin* in Swiss albino mice (Table 2) [116]. Furthermore, RAN with lime treatment induces global hyperphosphorylation of Rb (retinoblastoma) and histone H3 trimethylation modifications relax the chromatin. These epigenetic modifications deregulate Rb-E2F1 pathway and causes the up-regulation of proto-ongenes, *E2F1* and *securin* that may mediate oral carcinogenesis [117]. Promotor DNA hypermethylation of *BEX1* and *LDOC1* tumor suppressor genes and the consequent hyperactivation of NF-κβ signalling pathway is linked to the oncogenic effects of combined BQ and tobacco use that leads to increasing OSCC occurrence [118].

## 8 Conclusion

In summary, accumulating research evidence suggests that BQ and AN chewing with or without tobacco can induce OPMDs. Persistent AN and BQ chewing with OPMD conditions portend the oral or oro-pharyngeal cancer development. In presence of nitrosating agents such as nitrosonium ion (NO^**+**^) and nitrogen sesquioxide (N_2_O_3_), under the prevailing physiological conditions at stomach, the major alkaloids in areca nut and tobacco exhibit high propensity for the carcinogenic nitrosamines formation. Ingested as well as endogenously formed *N*-nitrosamines are, most likely, activated by metabolic phase-I metabolic enzymes to exert their mutagenic and carcinogenic effects. In addition, tobacco matter (smokeless tobacco) chewed along with BQ and to a lesser extent tobacco smoke also contains hydrophilic direct alkylating agents that can be easily absorbed by saliva that may elicit potent adverse health effects leading to the development of oral and esophageal cancers. The deleterious interaction of metabolically activated *N*-nitrosamines and direct alkylating agents of areca nut and tobacco with DNA entails genetic aberrations *viz*., aberrant DNA mutations, deregulated hyper- or hypomethylations of DNA and dysfunctional histone modifications. The genetic and epigenetic factors cumulatively influence the development of neoplasms. Accumulation of sporadic genetic and epigenetic lesions in the cellular signalling and regulatory pathways due to long-term BQ (with or without tobacco) chewing and tobacco use culminates into the development of head and neck cancers.

Our knowledge on the carcinogenesis of BQ and AN use has considerably evolved over past two decades, yet much remains unknown. Comprehensive data on the wide range of ingredients employed for BQ preparations, and diverse combinations of BQ usage patterns is warranted in order to understand and elucidate the genetic and epigenetic abnormalities associated with oral and oro-pharyngeal neoplasia. Although the endogenous formation and the corresponding metabolic activation of TSNAs is relatively well-known phenomenon, however, the molecular mechanisms of the endogenous formation of ASNAs, metabolic activation of ASNAs, and the concomitant DNA adducts formation propensity remains to be elucidated.

Lately, non-targeted analysis of DNA adducts (DNA adductomics [119]) and nucleic acid modifications (nucleic acid adductomics [120]) are emerging as powerful and versatile tools for comprehensively detecting both expected and unexpected DNA adducts and nucleic acid modifications using high resolution mass spectrometry. Ultimately, these adductomics approaches will provide us with a more comprehensive and in-depth understanding of the carcinogenic mechanisms underlying BQ and AN use.

## Funding

R.B. Muthukumaran: Project Grant, BT/PR24211/NER/95/715/2017 - DBT, New Delhi, India; Advanced State Biotech Hub Grant to Mizoram University BT/04/NE/2009 - DBT, New Delhi, India; P. Bhattacharjee: Project Grant, BT/PR24211/NER/95/715/2017 - DBT, New Delhi, India; N.S. Kumar: Advanced State Biotech Hub Grant to Mizoram University BT/04/NE/2009 - DBT, New Delhi, India; M.R. Chao: Grant number, 10.13039/100007225MOST 109-2314-B-040-018-MY3, the Ministry of Science and Technology, Taiwan; C.W. Hu: Grant number, 10.13039/100007225MOST 111-2628-B-040-005, the Ministry of Science and Technology, Taiwan.

## Declaration of Competing Interest

The authors declare that they have no known competing financial interests or personal relationships that could have appeared to influence the work reported in this paper.

## Data Availability

No data was used for the research described in the article.
